# “Just clearly the right thing to do”: perspectives of correctional services leaders on moving governance of health-care in custody

**DOI:** 10.1108/IJOPH-08-2023-0052

**Published:** 2024-07-05

**Authors:** Katherine E. McLeod, Amanda Butler, Ruth Elwood Martin, Jane A. Buxton

**Affiliations:** Department of Family Medicine, McMaster University, Hamilton, Canada; School of Criminology, Simon Fraser University, Burnaby, Canada; School of Population and Public Health, The University of British Columbia, Vancouver, Canada

**Keywords:** Health in prison, Correctional health-care, Prison staff, Health policy, Equivalence, Prison medicine

## Abstract

**Purpose:**

Governance models are a defining characteristic of health-care systems, yet little research is available about the governance of health-care delivered in correctional facilities. This study aims to explore the perspectives of correctional services leaders in British Columbia, Canada, on the motivations for transferring responsibility for health-care services in provincial correctional facilities to the Ministry of Health, as well as key lessons learned.

**Design/methodology/approach:**

Eight correctional services leaders participated in one-on-one interviews between September 2019 and February 2020. The authors used inductive thematic analysis to explore key themes. To triangulate early effects of the transfer identified by participants the authors used complaints data from Prisoners’ Legal Services to examine changes over time.

**Findings:**

The authors identified four major themes related to the rationale for this transfer: 1) quality and equivalence of care, 2) integration and throughcare, 3) values and expertise and 4) funding and resources. Facilitators included changes in the external environment, having the right people in the right places, a strong sense of alignment and shared goals and a changing culture in corrections. Participants also highlighted challenges, including ongoing human resourcing issues, having to navigate and define shared responsibilities and adapting a large bureaucracy to the environment in corrections. Consistent with outcomes described by participants, data showed that a lower proportion of complaints received after the transfer were related to health-care.

**Originality/value:**

The perspectives of correctional leaders on the transfer of governance for health-care services in custody to the community health-care system provide novel insights into the processes and potential of this change.

## Introduction

Governance of health-care services in correctional facilities is increasingly recognized as a critical component of addressing population health both in custody and in the community (McLeod *et al.*, 2020). Correctional services are essential partners in enabling change in governance and in the ongoing delivery of health-care services. However, the current literature holds a limited perspective of correctional services on governance models that have transferred responsibility for health-care services delivered in custody from the ministry responsible for corrections to the ministry responsible for health. The purpose of this study is to explore the rationale for this transfer of responsibility in British Columbia (BC), Canada, as well as key lessons learned from the perspective of correctional services leaders.

People who experience incarceration have a high burden of physical and mental health conditions and complex health-care needs (Fazel and Baillargeon, 2011; Fazel *et al.*, 2016). The intersection of social and structural inequities means that both before and after incarceration people face barriers to accessing health-care in community and are less likely to have a primary provider compared to the general population (Green, Foran and Kouyoumdjian, 2016; Fahmy *et al.*, 2018; Redmond *et al.*, 2020). Missed opportunities for prevention and early diagnosis, treatment and follow-up may allow health conditions to deteriorate and the higher costs of treating advanced or worsened conditions are often borne by community health-care systems, as are the public health consequences of undetected or untreated conditions.

In most jurisdictions worldwide, health-care services in corrections are under the mandate of the entity responsible for correctional facilities, such as the Ministry of Justice. However, in a growing number of jurisdictions, health-care services delivered in custody are being shared with or moved to, the entity responsible for health-care in the general population (such as the Ministry of Health). This transfer to health ministries is aligned with recommendations by the World Health Organization, and the United Nations’ Nelson Mandela Rules (United Nations General Assembly, 2015). Arguments in favour of health ministries being responsible for health-care in custody include improving clinical independence and practice standards and supporting integration with community health-care services. (Cinamon and Bradshaw, 2005; Enggist, 2013; Pont *et al.*, 2018; McLeod *et al.*, 2020) However, globally, there has been limited research about the process or impact of this change in governance (McLeod *et al.*, 2020).

In Canada, people who are on remand waiting for trial or sentencing and people who have been sentenced to less than two years are held in correctional facilities managed by the province or territory. People sentenced to two years or more are held in federal correctional facilities. Prior to the transfer, health-care services in BC’s 10 provincial correctional facilities were delivered by a private, for-profit company contracted by the Ministry of Public Safety and Solicitor General. On 1 October 2017, health-care services transferred to the provincial health services authority (PHSA), under the Ministry of Health. Stated aims of this change included improving service delivery in custody and strengthening continuity of care with community services (Correctional Health and BC Mental Health and Substance Use, 2017).

Limited research available about transferring governance for health-care services in correctional facilities to the community health-care system has primarily highlighted perspectives of health-care leadership and providers (Public Health England, 2016; McLeod, 2021). This study aims to provide new insight to the body of literature by highlighting the perspective of correctional leaders on this governance change.

## Methods

To explore perspectives of correctional services leadership, we conducted one-on-one interviews with Wardens (the individuals responsible for the overall operation and management of an institution, also known as Superintendents) and BC Corrections leadership, including directors, managers and other positions between September 2019 and February 2020. This study complements perspectives of health-care leadership on the transfer in BC explored in a contemporary study (McLeod, Buxton and Martin, 2023). We used purposive sampling to interview individuals with the greatest familiarity and knowledge of the transfer. BC Corrections provided a list of 13 leaders involved in the transfer. KM sent invitations to participate via email to all potential interviewees identified. Four people did not respond to this invitation, plus one follow-up invitation. One person declined to participate. Malterud *et al.* (2015) propose that the sample size in qualitative interview studies can be guided by “information power” which depends on the aim of the study, the sample specificity, the use of established theory, the quality of dialogue and analysis strategy. The narrow focus of the aim of the study allowed us to establish sufficient information power with a smaller number of participants by engaging those with highly specific knowledge. Interview questions focused on the process and early outcomes of the transfer and are provided in the Appendix.

The lead author (KM) conducted all eight interviews by phone or in person depending on the participant’s preference and location. At the time of the interviews, KM was a PhD student and had no previous relationship with any interviewees. Interviews were audio recorded and transcribed *verbatim*. De-identified transcripts were checked against the audio recording and each participant was assigned a two-letter identifier. We did not conduct any repeat interviews. Participants were given the option to review a copy of their transcript and a draft manuscript as form of member-checking. Four participants requested the transcript from their interview and seven were sent a draft manuscript for comment. Approval for this study was granted by the University of BC Behavioral Research Ethics Board (*H17* – 02577), the BC Mental Health and Substance Use Services Research Committee and by BC Corrections.

We conducted an inductive thematic analysis (Creswell and Creswell, 2018) of transcripts using NVivo 12 software to organize the data. We developed an initial code book using Kotter’s eight-step model of change management (Kotter, 2021) and existing literature on the rationale for this transfer of governance for health-care in correctional facilities.

All authors independently coded the same two transcripts and then came together to share and discuss codes adding those that had emerged from the data and collaboratively refining the code book. Using this revised code book, two authors (KM and AB) coded the remaining transcripts, adding and revising codes. Based on the final coding, all authors iteratively discussed and developed themes until consensus was reached.

To triangulate the early effects of the transfer identified by participants, we requested complaints data from Prisoners’ Legal Services. Prisoners’ Legal Services is operated by the West Coast Prison Justice Society. It provides legal aid (other than appeals) to people incarcerated in federal and provincial correctional facilities in BC. Two authors (KM, REM) learned about this data, and the volume of calls related to health-care, during a presentation to an undergraduate class on prison health. We examined data from calls received by the organization from people in provincial custody from 31 December 2015 to 31 December 2019. Details of each complaint received, including codes identifying the topic of complaint are recorded for administrative purposes by the staff member who takes the call. Any complaint which included the code “medical”, “mental health” or “medical – college complaint” was considered health-care-related. Complaints which contained none of these codes were treated as non-medical complaints. We examined the proportion of health-care complaints by year and conducted a bivariate logistic regression analysis using *R*.

## Findings

Eight members of correctional services leadership (five men and three women) participated in interviews. Three participants were employed as Wardens, and five held other leadership positions within BC Corrections. Interviews lasted an average of 49 min (range: 36–59 min).

**Figure 1:**
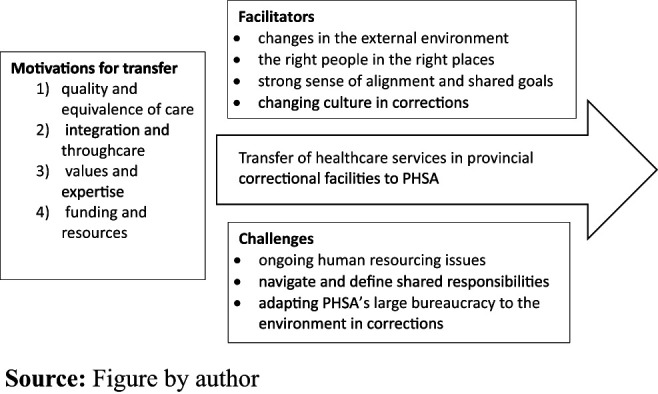
Themes identified in interviews with BC Corrections leadership about the transfer of health-care services in provincial correctional facilities to the provincial health services authority

We identified four major themes in participant descriptions of the rationale or motivations of the transfer of health-care services to PHSA: quality and equivalence of care, integration and throughcare, values and expertise and funding and resources (Figure 1). All four themes intersected with an overarching theme of a desire to improve access to and quality of health-care services and to contribute to better health outcomes for people in custody.

In discussing the process of the transfer, BC Corrections leadership identified facilitators, including changes in the external environment, having the right people in the right places, a strong sense of alignment and shared goals and a changing culture in corrections. They also highlighted challenges, including ongoing human resourcing issues, having to navigate and define shared responsibilities and adapting PHSA’s large bureaucracy to the corrections environment. Consistent with the perception described by interviewees, data from Prisoners’ Legal Service showed that following the transfer to PHSA, there was a reduction in health-care-related complaints.

### Quality and equivalence of care

Many participants framed the need for the transfer as a moral imperative to address the fact that people in custody have greater health-care needs but experience more barriers and receive lower-quality care due to the structure of health services in custody and disconnect from the community health-care system:

When you have a healthcare system that is driven by a finite budget and a bottom line, what ultimately happens is there’s substandard healthcare. And that’s what we were finding that individuals in our care were not receiving the same amount of care, or the same level and kind of care that others in the province were afforded. (TS)

This disparity was felt to be exacerbated in BC because prior to the transfer, health-care was provided by a private, for-profit company. Part of this moral argument was that health-care services in custody should be equivalent to health-care delivered in the community. Participants believed that moving services to PHSA would better enable community standards of care in custody since PHSA was an existing provider of health-care in the community. All health-care services delivered in community must be accredited by Accreditation Canada. Health-care services in custody had previously been excluded from this legislated requirement and participants highlighted how the transfer enabled correctional health-care services access to these structural levers to raise standards of care:

And PHSA came with a whole accreditation, right. And the centres aren’t accredited yet, but their approach to business is from an accredited lens and they know healthcare. They’re the experts. And I can defer to them and rely on them to advocate for us. (DC)

Correctional service leaders highlighted how working towards raising standards of care had resulted in training opportunities and improved support for health-care staff, as well as a shift in the culture and perceived professionalization of health-care:

I can see the professionalization of the department, just how it looks and how it sounds. But I think from the floor, I think it’s just more of that– the nurses are more willing to talk more with the people in custody about their problems, that sort of thing. Just a little more of that kind of going on and provide that level of medical type care, I think is what the improvement there has been. (PW)

The recognition and status of PHSA was also described as attracting high-quality candidates for health-care positions, which contributed to raising standards of care to be consistent with services delivered in the community.

Some participants expressed a belief that improved health-care would impact security and correctional outcomes, such as recidivism. They described an expectation that with better health-care people would be less likely to return to custody after release. One participant (DC) gave the example that improved health-care services for substance use, including the elimination of the waitlist for medications for opioid use disorder, could improve health and safety within correctional facilities themselves:

A huge vulnerability for government if we’re not even able to provide opioid replacement in custody. You know, that results to contraband getting into the centre and deaths and assaults. Not just on inmate on inmate but [also] on staff. (DC)

### Integration and throughcare

Participants consistently described a central rationale for the transfer to PHSA in terms of better continuity of services between custody and community. Previously, correctional health-care, including medication, ended at the doors of the facility. Participants described this disruption as contributing to poorer health and legal outcomes:

So people would come in and be in drug withdrawal and then […] we would work to get them on the protocol. And then when they get released if they didn’t have a service provider physician […] that can be really difficult and dangerous for the first couple weeks after they get out. So I think PHSA having those links in the community and setting the inmates on release, I think, has been better. And I think that’s helped. (NL)

Participants provided examples of tangible improvements in continuity of care that had already occurred, including providing people with medication to take with them on release. Integration with the larger health-care system was also thought to be an opportunity to strengthen advocacy and resourcing in the community that could help divert people from custody and into appropriate services. Participants described the status of PHSA as increasing awareness of health-care in corrections within the broader health-care system and an opportunity to prioritize needs, address gaps and improve care:

And maybe we’ll see more resources in the community because with Corrections Health at the table they’re able to tell PHSA and greater Ministry of Health the gaps in healthcare. (EH)

Participants also hoped that increased resources of PHSA would help to support people with high mental health and substance use needs, though acknowledged that human resourcing was an ongoing challenge:

Without knocking a contractor, it was just giving the inmate immediate care with no follow-up after. And it was unfair. I mean, they were at a disadvantage. They would then steal for their addictions. We didn’t follow-up on addiction. So those are kind of basic things that I can’t give you numbers to, but I do know that when you give someone a chance, better care, they don’t need to refer to crime to be able to survive. (JF)

Correctional leaders felt that PHSA had status within the health-care system as well as connections and access to services that could facilitate better continuity of care, including improving the flow of health information and facilitating follow-up with community providers more effectively.

### Values and expertise

Participants highlighted the transfer of responsibility for health-care services in correctional facilities to PHSA represented a better alignment of responsibilities with appropriate expertise. No longer having to manage oversight of contracted health-care services meant that correctional leadership at all levels could allocate their time to the non-health-related correctional services and security:

It just kind of makes business sense when you already have a whole ministry and health authorities of people who are used to managing health to let them manage the health rather than to have people who are correctional experts managing health. (GA)

Similar to the increased access to health human resources, BC Corrections leadership also identified PHSA as having a greater wealth of experts in health and health-care services that would facilitate improved quality of care in custody:

Just with Ministry of Health being the experts, they’re the experts in that business as opposed to Corrections hiring out an organization, private contractor, that may have varying levels of degrees of expertise and likely not have the same number of healthcare advisors. (NL)

In addition to improving care, partnering with experts in health-care was thought to raise the credibility and recognition of correctional services and of the health-care services delivered in custody:

I think just to have a really recognized partner, established partner, of that professional magnitude was important for BC Corrections to be seen as our healthcare provider […] So I think as BC Corrections we thought, you know what, this is where we want to be seen as– we’re professionalized in what work we do as Corrections. It’s been a long haul but we’re trying our best to show and let people know it’s not what you see in the movies right. (PW)

Participants identified a shift in the culture of corrections that both enabled and was fostered by the transfer of health-care services to PHSA:

I think it’s even professionalized our deputy provincial directors and the wardens and stuff. We’re not that big, really. BC Corrections isn’t that big in comparison to something like PHSA. So I think it’s kind of helped us realize the potential or executive decisions they impact– and how you can as an organization be healthy and work at that level, the higher level. I think that’s something that’s kind of a byproduct of all this. (PW)

BC Corrections leaders also expressed greater alignment of their organization’s values with PHSA than they had experienced with previous health-care contractors, such as evidence-based decision-making and shared objectives:

We’re just so aligned in our thinking and our ultimate objective is to provide the best care for those that are in custody. (DC)

An example cited by several participants was the elimination of the waitlist for medications for opioid use disorder. Ultimately, when asked for advice they would give other jurisdictions considering a similar change in governance model, most participants expressed an overall positive view of the transfer and said they would recommend that other jurisdictions make the change.

### Funding and resources

PHSA’s access to funding, personnel and resources were thought to be key facilitators of higher standards of care and improved linkages with community services. Part of the benefit described by correctional leaders was a new alignment of goals in working with another publicly funded body. This was contrasted with the contractor model in which profit margins were drivers of care decisions:

The previous contract model, it all comes down to the profit that the contractor can make out of this […] We’re not dealing with that with PHSA. We’re talking about another public body who is committed to delivering a service and meeting objectives similar to what BC Corrections is doing. And so you’re not constantly up against that challenge, ‘well, this will cause extra resources and reduce our bottom line’. (CE)

Participants highlighted the stability of health-care resources that PHSA brought, in contrast to the cycles of contracts with different service providers. Participants also discussed the greater financial resources that PHSA was able to command in providing care for people in custody, including paying for equipment and staff. Most participants spoke of increased access to health-care resources through PHSA, particularly specialists and experts such as in pharmacy and infection control. Though mostly positive, these additional resources could also present a challenge since they were sometimes viewed as being ill-adapted to the carceral environment:

So there’s more resources in terms of, you know, their infection control. They have a gazillion people in PHSA, it’s beyond me. They, you know, all of a sudden infection control takes on a whole other meaning […] Whereas our contractor previously they had an expert, but then they would have to reach out further to CDC or whatever. (EH)

Finally, PHSA introduced telemedicine to the delivery of service in custody which expanded access to both physician and specialist resources. This was seen as particularly important for centres located in the north and other remote or rural areas in which correctional facilities had consistently struggled with adequate access to physician services.

### Lessons learned

Participants described several key facilitators and challenges in the process of the transfer (Figure 1). Though these lessons reflected elements of the Kotter model of change, it was not a linear process. Rather, facilitators and challenges described illustrated a dynamic and iterative movement of change. Examples and illuminative quotes are provided in Table 1.

Key facilitators included: changes in the external environment, having the right people in the right places, strong sense of alignment and shared goals and a changing culture in corrections.

Participants highlighted the timing of the change in the context of the external environment, including changes to public and political perceptions of and interest in incarcerated populations. Participants explained that although correctional leaders in BC had been advocating for this change for years, the Ministry of Health had been unwilling to take on responsibility for care in corrections. This shift was credited to the growing number of calls for action in reports and investigations, changing public perception about population health and the will of individuals in key leadership positions.

The importance of having the right people in the right places was highlighted frequently by participants. This included the will and motivation of decision makers to initiate the change, as well as leadership within both health-care and corrections in tackling challenges and moving work forward throughout the transfer process. Having the right people in the right places resulted in alignment and shared goals between corrections and health, which participants described as key facilitators of the transfer. The transfer took place over less than a year, and participants believed that this was possible through the collaborative approach and alignment that both sides held to solve problems and move work forward. The establishment of joint committees to tackle elements of the change were seen as key facilitators. Finally, as both a facilitator and a result of the change, participants described a changing culture internal to corrections that is working towards person-centred approaches and a focus on the long-term trajectory of people who experience incarceration.

Participant descriptions of the transfer process and the ongoing relationship between PHSA and BC Corrections also illuminated several challenges, including ongoing human resourcing issues, having to navigate and define shared responsibilities and adapting PHSA’s large bureaucracy to the environment in corrections. Challenges with human resources were described both in terms of difficulties in the processes of PHSA hiring the entire frontline workforce, as well as ongoing difficulties with recruitment, particularly for rural centres. Another complex process was navigating and defining shared responsibilities within the context of this partnership. For example, for a person in custody to attend an appointment in the community requires security escorts which has implications for BC Corrections staffing.

Similarly, some participants described differences in views about the amount and type of health information that might be relevant to security and safety of staff and people in custody. Participants explained that navigating these challenges was made difficult by the large bureaucracy of PHSA and their lack of experience in correctional centres. Participants described instances where they had to educate PHSA about the realities of the environment and address assumptions about corrections.

### Complaints data from prisoners’ legal services

**Figure 2:**
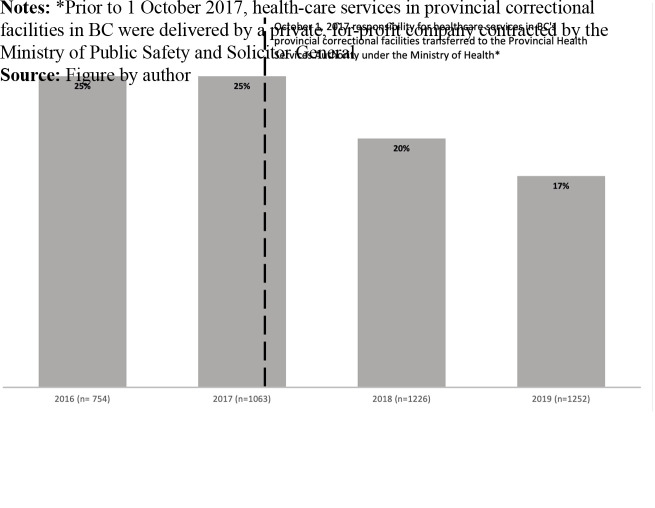
Proportion of complaints received by Prisoners’ Legal Services from people incarcerated in provincial correctional facilities in British Columbia which concerned health-care by year, from 1 January 2016 to 31 December 2019

Participants described that an early impact of the transfer to PHSA had been a reduction in the number of complaints about health-care services. Using data on complaints received by Prisoners’ Legal Services we found that despite an increase in the overall number of complaints received (attributed to additional staffing and the resulting increase in capacity at Prisoners’ Legal Services) the proportion of complaints related to medical services has declined since 2017 (Figure 2). In bivariate analysis, complaints in 2018 and 2019 were significantly less likely to be medical compared to 2016 (OR 0.75 95%CI 0.61–0.93 and OR 0.63 95%CI 0.51–0.79, respectively).

## Discussion

BC Corrections leadership described several motivators and benefits for transferring health-care service delivery in provincial correctional facilities to the BC Ministry of Health. Participants also identified several key elements that acted as facilitators to the transfer process as well as challenges that the two organizations worked through and, in some cases, continued to navigate. These experiences provide valuable insights and lessons learned that may be applicable to other jurisdictions looking to transform governance structures for health-care services delivered in corrections.

Our study provides novel insights into, and specific examination of, the process and outcomes of a transfer of health-care services to the organization responsible for health-care in the community from the perspective of correctional leadership. A small number of studies have included the perspectives of corrections leadership and policymakers in exploration of key principles of governance and health-care in custody, including the principle of equivalence and its application in custody (Bretschneider and Elger, 2014; Ismail and de Viggiani, 2018). Studies have also examined experiences and impacts of this type of transfer from the perspective of health-care services. These described many of the themes of benefit identified in our study including improved resourcing and increased health-care personnel, increased quality of care, improved access to services and a wider range of services offered, strengthened connection to community services. There were also similarities in persistent difficulties such as with recruitment and retention of health-care staff, inadequate resourcing and prejudice within health care and the general public with regard to people who experience incarceration. (Department of Health and International Centre for Prison Studies, 2004; Cinamon and Bradshaw, 2005; Hayton and Boyington, 2006; Royal College of Nursing Scotland, 2016; Leaman *et al.*, 2017; Bengoa *et al.*, 2018; Accreditation Canada, 2020; WHO Regional Office for Europe, 2020; McLeod, Buxton and Martin, 2023). The alignment of motivations and outcomes for the transfer identified by leadership in both corrections and health-care across jurisdictions indicate that this governance model may present opportunities to leverage mutual benefit and shared aims. In this study, Corrections leadership also provided novel perspective on the process and impact of developing this type of integrated partnership for correctional services. These included the challenges of introducing a health-care organization to the constraints and realities of the corrections environment, the importance of communication in developing shared aims and addressing preconceptions and the potential benefits for correctional organizations themselves.

While the context of Canada’s universal public health insurance system may not be globally generalizable, key learnings around the economics of expertise and resources shared across sectors may be applicable in a variety of governance and delivery models. In this study increased resourcing was seen as strengthening both organizations. In addition to the internal resources of PHSA as an organization, resourcing was improved by an increased budget dedicated to providing health-care services in custody. In both BC and in Alberta, where the transfer to Alberta Health Services occurred in 2010, there was a reported 40% increase in per-capita spending for health-care services delivered in custody (The Expert Advisory Committee on Health Care Transformation in Corrections, 2019). Lessons from the UK experience suggest that continued investment in prison health-care is critical to ensuring sustained benefits from transferring responsibility to the ministry of health. The complete transfer of commissioned services in prison to the NHS in 2006 was followed by a significant investment in services, including evidence-based mental health and substance use care and universal vaccine programmes (Piper *et al.*, 2019). However, austerity measures in the years following led to major staffing shortages and recruitment challenges, and a presumably related increase in poor health outcomes including mortality, self-harm and hospitalizations (HM Inspectorate of Prisons for England and Wales, 2018).

Participants discussed the transfer and quality improvements in terms of achieving equivalence with community services. Across jurisdictions and governance models, equivalence of services with communities is complicated by the realities that unequal and inequitable access to, and quality of health-care services is also experienced in the community, even in systems with universal health insurance, through intersecting social and structural determinants including (but not limited to) income, stigma and racism (Olah, Gaisano and Hwang, 2013; Henderson *et al.*, 2014; Fahmy *et al.*, 2018; M.E. Turpel-Lafond (Aki-Kwe), 2020). Acknowledging these tensions, equivalence provides a tangible benchmark against which to measure improvements to care in custody.

Participants highlighted the importance of individual leaders, strong communication and collaboration and culture change as key facilitators of the transfer. These identified essential elements are consistent with Kurt Lewin’s theories of group dynamics and field theory, which underpin most models of planned change (Burnes, 2004; Antwi and Kale, 2014). The transfer and transformation of health-care services in custody through a change in governance model is complicated by the ongoing shared responsibilities and power dynamics between corrections and health-care services. This study reveals that the leadership and culture of each organization may play a fundamental role in influencing the staff of both health and corrections and in actualizing change.

Participants identified reduced complaints as an early outcome of the transfer and an anticipated measure of improvements of quality. The reduction in complaints was demonstrated in data provided by Prisoners’ Legal Services. This verification may provide a level of confidence in the outcomes participants expressed that they believed had resulted from the transfer despite an absence of formal evaluation. Future research is needed to understand how governance structures affect patient experiences of services and health-care needs.

### Limitations

We used purposive sampling to interview BC Corrections leadership who had been involved in the transfer and would thereby be most knowledgeable of the transfer process. Given this investment, it may be that participants could have been more likely to take a positive view of the process and its outcomes than frontline staff or less engaged members of leadership. However, participants candidly identified and discussed challenges, so we do not have reason to believe negative views were intentionally omitted. Additionally, we interviewed people at multiple levels of leadership with varying degrees of ownership or accountability for the transfer. Member checking, through participant review of their own transcript and the draft manuscript, provided opportunities to identify gaps. Though interviews were conducted less than two years after the transfer, this temporal proximity was a strength in that experiences were fresh and, in many cases, ongoing. However, this limited our ability to explore long-term and sustained outcomes of the transfer and may potentially reflect optimism associated with the change. Future research should seek to understand challenges and outcomes in the medium and long term. Finally, data about complaints was collected as part of the administrative work of Prisoners’ Legal Services and not for the purpose of research or evaluation. This may have affected how complaints were coded. To support rigor in this analysis, we applied a conservative definition of “health-care-related” complaints and counted only those explicitly labelled as medical or mental health complaints.

## Conclusions

Insights and experiences of leadership in BC Corrections about the transferring of health-care services to PHSA provide lessons learned for jurisdictions working to improve care in custody regardless of governance structure. Increased integration and collaboration between health-care services delivered in custody and the community may have a positive effect on health-care, health outcomes, as well as both health and correctional organizations.

## Figures and Tables

**Table 1:** Kotter’s eight steps of change management were used to explore the process of the transfer, not as a linear process as described in the model but to highlight the role of facilitators and challenges as factors contributing to change

Kotter’s eight steps of change management	Key lessons learned (facilitators and challenges)	Illustrative quotes
*Create a sense of urgency*	External environment played a role in creating change, including public perceptions and political will	It took us a better part of 10 years to have the political will to get here. But we just kept at it. We kept at it and we kept at it and we advocated and we kept talking to people. And kept trying. And then, you know, I don’t want to say we got lucky. But there is, you know, there was an element of okay, the will is here now. (TS)
*Build a guiding coalition*	Having right people in the right places was identified by participants as a key facilitator both of gaining political will for change and of driving the transfer process forward	And so we were a team from the get-go. There was nobody– like, yeah we knew who was from PHSA and who was from Corrections. But it was very cohesive. This is the team. This is what we got to do. Let’s get there together. (EH)
*Create a vision and a strategy*	Negotiating needs and shared responsibilities was an ongoing challenge in establishing vision and strategy. Orienting PHSA to corrections and address preconceived notions affected the process and development of a shared vision and strategy	There was definitely an education process that maybe some of the PHSA project folks came in with some assumptions that we knew very clearly weren’t going to be feasible. And so a lot of that just has to do with the realities of working in a correctional health environment that doesn’t fit with the usual health world. (GA)
*Communicate the vision*	Ongoing communication was identified as a key component of maintaining and actioning alignment	I mean, the communication plan, that’s probably the biggest thing… there was an in-person meeting structure. There was documentation structures. There was conference calls. So they hit the communication very, very well. And I– like I say, it was seamless. It was very, very well done. (JF)
*Remove obstacles*	Participants described having to work through challenges of introducing a large bureaucracy to a new environment	PHSA is also a big massive body and I think sometimes there can be– I don’t want to call it strained, but sometimes the momentum falls a little bit if we’re dealing with departments that are outside of the Correctional Health Services core team. (GA)
*Create short-term wins*	Participants highlighted outcomes that marked early successes of the transition	So then you’re going to look at successes, if you measure complaint forms from when [the contractor] was in there, for and then you measured complaints now, I bet you you’d see a reduction. (JF)
*Consolidate gains and produce more change*	A new culture of metrics and quality improvement was seen as a way of recognizing successes and moving improvement work forward	One of the areas of focus was developing metrics to measure service delivery and outcomes moving forward so that we can kind of measure how we are doing. (CE)
*Anchor new approaches in culture*	Some participants highlighted a broader change in the culture of corrections as both a result and component of the transfer of health-care services to PHSA.	We’re going through a culture change right now on the Corrections side where people are– staff are expected to see the individuals that are in custody as people that are in their care. Not as– they’re not just with us to supervise. They’re with us because we need to care for them. (DC)

**Source:** Table by authors

## Appendix. Interview script

*Context (Decision)*

1. Please would you tell me about your role and how it relates to the change in responsibility for health-care delivery for people in BC correctional facilities?
2. What is the story of how BC arrived at the decision that PHSA would assume responsibility for health-care services in correctional facilities?
  - What were the motivators?
  - What were the goals?
  - Please would you share a key moment that you feel moved the idea forward or that set the idea back?
  - Were there any other key moments you could share?
3. What was the process of moving from the decision to actually transferring responsibility to PHSA in October?
  - What were your expectations?
  - Were they met?
4. From your perspective, what were some of the challenges in the transfer process?
  - How were they addressed?
  - What are some anticipated challenges moving forward?
5. What were some of the important facilitators of the process?
  - What will be the facilitators moving forward?

*Expectations/changes*

6. From your perspective, what are the biggest changes so far that have resulted from the transfer of responsibility for service delivery?
  - What did you anticipate would be the changes [what were your expectations?]
    – Were they met?
    – What surprised you?
  - What were or are facilitators and barriers to the transfer’s impact?
7. From your perspective, are there further expected effects of PHSA assuming responsibility for service delivery? [short term? long term?]
  - What do you expect will be other outcomes of the transfer?
  - In what ways do you anticipate the transfer will change the continuity of care?
8. What does the success of this transfer look like to you?
  - What does Corrections view as success?
  - What would you say success looks like for the population?
  - What does the Ministry of Health regard as success?
9. How is the quality of health-care services defined in this context?
  - In what ways do you anticipate that the transfer will impact quality?
  - How is quality of health care measured?
    – Are there indicators?
    – Quality improvement?
  - Are there aspects of quality that are important but not captured in current data/monitoring?
10. Is there anything else you’d like to add?

*Source*: By authors
